# Primary mental health prevention themes in published research and academic programs in Israel

**DOI:** 10.1186/2045-4015-4-3

**Published:** 2015-02-23

**Authors:** Ora Nakash, Liat Razon, Itzhak Levav

**Affiliations:** School of Psychology, Interdisciplinary Center (IDC), P.O. Box 167, Herzliya, 46150 Israel; Department of Community Mental Health, Faculty of Social Welfare & Health Sciences, Haifa University, Haifa, 31905 Israel

**Keywords:** Primary prevention, Mental health education, Mental health research, Israel

## Abstract

**Background:**

The World Health Organization Comprehensive Mental Health Action Plan (CMHAP) 2013–2020 proposes the implementation of primary prevention strategies to reduce the mental health burden of disease. The extent to which Israeli academic programs and published research adhere to the principles spelled out by the CMHAP is unknown.

**Objective:**

To investigate the presence of mental health primary prevention themes in published research and academic programs in Israel.

**Methods:**

We searched for mental health primary prevention themes in: (1) three major journals of psychiatry and social sciences during the years 2001–2012; (2) university graduate programs in psychology, social work and medicine in leading universities for the academic year of 2011–2012; and (3) doctoral and master's theses approved in psychology and social work departments in five universities between the years 2007–2012.

We used a liberal definition of primary prevention to guide the above identification of themes, including those related to theory, methods or research information of direct or indirect application in practice.

**Results:**

Of the 934 articles published in the three journals, 7.2%, n = 67, addressed primary prevention. Of the 899 courses in the 19 graduate programs 5.2%, n = 47, elective courses addressed primary prevention. Of the 1960 approved doctoral and master's theses 6.2%, n = 123, addressed primary prevention. Only 11 (4.7%) articles, 5 (0.6%) courses, and 5 (0.3%) doctoral and master's theses addressed primary prevention directly.

**Conclusions:**

The psychiatric reform currently implemented in Israel and WHO CMHAP call for novel policies and course of action in all levels of prevention, including primary prevention. Yet, the latter is rarely a component of mental health education and research activities. The baseline we drew could serve to evaluate future progress in the field.

## Background

One of the pressing public health challenges societies face is to reduce the burden of mental, behavioral and substance abuse disorders [1]. To address the challenge, the World Health Organization (WHO) Comprehensive Mental Health Action Plan 2013–2020 proposes the “*implementation of strategies for primary… prevention in mental health*” [2]. This policy decision recognizes both the expanding knowledge-base that now exists to support evidence based interventions [1, 3], and the need to cover the outstanding debt that the mental health systems have contracted with society regarding primary prevention. It is therefore not surprising that when a large panel of international experts was asked to rank the “grand challenges” in mental health (defined as a specific barrier that, if removed, would help to solve an important mental health problem) primary prevention was placed first among the 40 top grand challenges. This was above the identification of biomarkers (ranked 18^th^) and the redesign of the health systems (ranked 20^th^) [4].

Key professional international organizations support the agenda set by WHO. For example, the American Psychological Association recently published guidelines for primary prevention in psychology [5]. These guidelines "*encourage psychologists, including those within the policymaking process, to strive to engage in prevention practice, research, and education to enhance human functioning*" [5].

This (re)awakening of primary prevention in mental health services is also relevant in Israel, where the current mental health reform makes it even more compelling [6]. As part of this reform, the responsibility for the provision of mental health services has been transferred from the ministry of health to the four national health maintenance organizations. This policy decision poses an opportunity for a major multidimensional change in the way mental health services are conceived, programmed and delivered in the following key domains of mental health [7]: (a) programs and services of primary prevention constitute a human rights issue enshrined by different UN Conventions such as the one referring to the rights of the child; (b) the mental health needs of the population do not seem to abate with the use of current therapeutic means, despite the progress in evidence- based therapies; (c) findings from epidemiology and other disciplines (among others, child development, neurosciences, genetics) converge indicating that a set of modifiable conditions could be imputed for the causes of many mental disorders; (d) the costs of child maltreatment – as an example area for preventive action – are substantial, while the cost for mental health primary prevention interventions are rewarded by net savings; and (e) intervention programs exist and are of acceptable efficacy and effectiveness [5].

The US Institute of Medicine [3] proposed using Gordon’s [8] classic public health distinctions between primary, secondary and tertiary mental health prevention. Primary prevention includes the following: (a) *Universal primary prevention* is defined as those "interventions that are targeted at population groups that have not been identified on the basis of increased risk"; (b) *Selective primary prevention* includes interventions that "target individuals or subgroups of the population whose risk of developing a mental disorder is significantly higher than average, as evidenced by biological, psychological or social risk factors"; and (c) *Indicated primary prevention* that "targets high-risk people who are identified as having minimal but detectable signs or symptoms foreshadowing mental disorder, or biological markers indicating predisposition for mental disorder but who do not meet diagnostic criteria for disorder at the time" [3], pp. 22–24.

### Objective

The above introduction highlights the need to implement primary prevention strategies to reduce the burden of mental health disease. The extent to which the Israeli academic program and research agendas adhere to the principles spelled out by the CMHAP with regard to prevention is unknown. To answer this issue, we investigated the inclusion of mental health primary prevention themes in published scientific articles and educational programs in university settings in Israel.

## Methods

We used Gordon's [8] definition of primary prevention, that is comprised of three target areas: universal, selective and indicated [3]. We reviewed: (a) publications in three major Israeli journals of psychiatry and social sciences published from 2001–2012 (total: 934 articles. Letters, editorials and book reviews were excluded from the review). We reviewed the following journals: *The Israel Journal of Psychiatry and Related Sciences*, which is the leading mental health journal in Israel and includes publications in the fields of psychiatry, psychology and social work, and less often, on psychiatric nursing and occupational therapy *Trends (Megamot,* in Hebrew*)*, a major interdisciplinary journal in the social sciences, that includes publications in psychology, social work and education; and *Society and Welfare (Hevra Verevaha;* In Hebrew*),* which includes articles related to social welfare; (b) curricula of academic graduate programs in leading universities for the academic year 2011–2012 (N = 899 courses). We reviewed programs in psychology (n = 12), social work (n = 4) and medicine (n = 3); and (c) doctoral and master's theses (N = 1960) in psychology and social work in five universities from 2007–2012.

We collected the information directly from the websites (e.g., list of publications) and through direct contact with the academic secretaries when needed (e.g., comprehensive list of courses, list of doctoral and masters theses). We reviewed full abstracts of all papers included in this review and when in doubt about the relevance of the paper to mental health primary prevention (broadly defined) we reviewed the full text (approximately 5% of the publications). With regard to graduate program curricula, we first reviewed courses' titles to search for themes related to mental health primary prevention very broadly defined. For the identified relevant titles we reviewed the syllabus of the course (approximately 5% of courses). Similarly, we first reviewed the Masters' and Doctorate theses' titles to search for themes related to mental health primary prevention very broadly defined. For the identified relevant titles we reviewed the full abstract to determine their relevance to the topic (approximately 10%).

The collected information was first coded with reference to a topic related to primary prevention in mental health (yes/no) and subsequently, for the specific level of prevention (universal/selective/indicated). Lastly, we coded whether the item concerned primary prevention *directly* – by describing interventions that can be applied directly (e.g., prevention programs for mental health problems), or *indirectly* – by providing information on modifiable factors that could be used ultimately in primary prevention interventions in mental health (e.g., role of modifiable social factors for developing mental health problems; related topics such as stigma).

In coding the materials we followed recommendations made by Braun and Clarke [9] for using thematic analysis in psychology. To establish consensus reliability [9, 10], we followed the following process: the materials were first read and coded independently by the first two coauthors who then convened to discuss the coding assigned. When disagreement arose, the team met to discuss the source of disagreement and when needed, the third coauthor was consulted. To code, we used a liberal approach, erring in the direction of inclusiveness. We calculated percentages for each piece of information when the base was higher than five items.

## Results

### Publications in major psychiatric and social sciences journals

The journals of psychiatry and social sciences surveyed were: (1) *The Israel Journal of Psychiatry and Related Sciences:* Of the N = 500 research articles published from 2001 to 2012, n = 16 articles addressed primary prevention (3.2%), the majority of them indirectly (Table 1). Most of the identified articles referred to stigma and the familial and societal effects of chronic mental illness, as well as to the sequelae of exposure to traumatic events. One article addressed universal primary prevention directly (e.g., "Prevention of eating disorders: A review of outcome evaluation research"); four addressed universal primary prevention indirectly (e.g. "On stigma in our society"); nine, 1.8%, addressed selective primary prevention indirectly (e.g. "Children living under a multi-traumatic environment: The Palestinian case"); one addressed indicated primary prevention directly ("Prevention of recurrent postpartum depression - a reasonable option?"); and one article addressed indicated primary prevention indirectly ("Family variables related to behavioral problems in childhood").

**Table 1:** **Number and percentage of publications addressing primary prevention, both direct and indirect** ^**1**^ **in psychiatry and social sciences journals in Israel**

Journal	Universal primary prevention	Selective primary prevention	Indicated primary prevention
Israel journal of psychiatry	n: 5 articles (1.0%)	n: 9 articles (1.8%)	n: 2 articles
Reviewed articles: N = 500	Direct – 1	Direct – 0	Direct – 1
	Indirect/related – 4	Indirect/related – 9 (1.8%)	Indirect/related – 1
	Examples:	Example:	Example:
	Direct: Prevention of Eating Disorders: A Review of Outcome Evaluation Research	Indirect/related: Children Living under a Multi-Traumatic Environment: The Palestinian Case	Direct: Prevention of Recurrent Postpartum Depression - a Reasonable Option?
	Indirect/related: On Stigma in Our Society		
***Megamot*** (in Hebrew)	**n: 1 article**	**n: 6 articles (3.0%)**	**n: 2 articles**
Reviewed articles: N = 202	Direct – 0	Direct – 0	Direct – 0
	Indirect/related – 1	Indirect/related – 6 (3.0%)	Indirect/related – 2
	Example:	Example:	Example:
	Indirect/related: The role of social support, self-esteem and adaptation in explaining the tendency for suicidal thoughts among the youth	Indirect/related: Coping resources of children in dysfunctional families	Indirect/related: Psychological distress among adolescents form the West Bank who took an active part in the resistance to the Israel’s unilateral disengagement plan
***Hevra Verevaha*** (in Hebrew)	**n: 3 articles**	**n: 34 article (14.6%)**	**n: 5 articles (2.2%)**
Reviewed articles: N = 232	Direct – 0	Direct – 8 (3.5%)	Direct – 1
	Indirect/related – 3	Indirect/related – 25 (10.8%)	Indirect/related – 4
	Example:	Examples:	Examples:
	Indirect/related: The perception of consumers with chronic psychiatric illnesses in the Israeli public	Direct: Could it there a "bright future" for the children "red" alarm? Building resilience for parents and young children exposed to ongoing threats of terrorism in Sderot	Direct: Elem’s outreach vans: outreach service to the street youth
		Indirect/related: Distress and ideological isolation among teenagers who were evacuated or who opposed the evacuation of settlements: A two year follow-up	Indirect/related: The trip to the Far East: Israeli hikers and their experience with psychoactive substances

^1^Direct primary prevention refers to interventions that can be applied directly (e.g., prevention programs for mental health problems); indirect/related primary prevention refers to studies providing information on modifiable factors that could be used ultimately in primary prevention interventions.

2001–2012.

*(2) Trends (Megamot,* in Hebrew*):* Of the N = 202 articles published from 2001 to 2012, nine, 4.5%, addressed primary prevention (Table 1). Most articles referred to preventive work with children and adolescents. One article addressed universal primary prevention indirectly ("The role of social support, self-esteem and adaptation in explaining the tendency for suicidal thoughts among the youth"); six, 3.0%, addressed selective primary prevention indirectly (e.g. "Coping resources of children in dysfunctional families"); and two articles addressed indicated primary prevention indirectly (e.g. "Psychological distress among adolescents who took an active part in the resistance to the Israel’s unilateral disengagement plan").

*(3) Society and Welfare (Hevra Verevaha;* In Hebrew*):* Of the N = 232 articles published from 2001 to 2012, n = 42 (18.0%) dealt with primary prevention (Table 1). Most articles addressed selective primary prevention related to community work with civilians who were exposed to political violence or security threats, as well as risk behaviors among the young. Three articles addressed universal primary prevention indirectly (e.g. "The perception of consumers with chronic psychiatric illnesses in the Israeli public"); n = 8, 4.0%, addressed selective primary prevention directly (e.g. "Could there be a 'bright future' for the children that were exposed to the "red" alarm? Building resilience for parents and young children exposed to ongoing threats of terrorism in [the city] Sderot"); and n = 25, 12.5%, articles addressed selective primary prevention indirectly (e.g. "Distress and ideological isolation among teenagers who were evacuated or who opposed the evacuation of settlements: A two year follow-up"); one, addressed indicated primary prevention directly ("Elem's outreach vans: outreach service to the street youth"), and four articles indicated primary prevention indirectly (e.g. "The trip to the Far East: Israeli hikers and their experience with psychoactive substances").

### Curricula of academic programs in psychology and social work, and in medical schools

We reviewed the curricula in psychology (N = 260 courses in 12 graduate programs of five universities); social work (N = 268 courses in graduate programs in four universities); and medical schools (N=: 371 courses of three universities) during the academic years 2011–2012 (Table 2). Of the graduate programs in psychology, eight courses (3.0%) addressed mental health primary prevention. Many programs included no mental health primary prevention. All courses that did were elective and indirectly addressed selective primary prevention related to exposure to trauma and stressful life events (e.g., "Trauma – coping and consequences").

**Table 2:** **Number and percentage of courses offered in medical schools and graduate programs in psychology and social work in Israel addressing primary prevention, both indirect and direct** ^**1**^ **(Academic year 2011–1012)**

	Universal primary prevention	Selective primary prevention	Indicated primary prevention
Graduate programs in psychology			
**University I** ^**2**^			
Adult clinical psychology program	**0 courses**	**n: 1 course**	**0 courses**
Reviewed courses: N = 20		Direct – 0	
		Indirect/related – 1	
		Example:	
		Indirect/related: Trauma – coping and consequences	
Child clinical psychology	**0 courses**	**0 courses**	**0 courses**
Reviewed courses: N = 24			
Rehabilitation psychology	**0 courses**	**n: 1 course**	**0 courses**
Reviewed courses: N = 25		Direct – 0	
		Indirect/related – 1	
		Example:	
		Indirect/related: The consequences of disability	
**University II**			
Adult clinical psychology	**0 courses**	**0 courses**	**0 courses**
Reviewed courses: N = 23			
Child clinical psychology	**0 courses**	**n: 2 courses**	**0 courses**
Reviewed courses: N = 21		Direct – 0	
		Indirect/ related – 2	
		Example:	
		Indirect/related: Resilience of children under conditions of deprivation, trauma and violence	
**University III**			
Clinical psychology	**0 courses**	**0 courses**	**0 courses**
Reviewed courses: N =24			
Neuro-rehabilitation psychology	**0 courses**	**0 courses**	**0 courses**
Reviewed courses: N = 23			
**University IV**			
Adult clinical psychology	**0 courses**	**0 courses**	**0 courses**
Reviewed courses: N = 17			
**University V**			
Clinical psychology	**0 courses**	**0 courses**	**0 courses**
Reviewed courses: N = 22			
Clinical-educational psychology	**n: 3 courses**	**0 courses**	**0 courses**
Reviewed courses: N = 21	Direct – 0		
	Indirect/related – 3		
	Example:		
	Indirect/related: The educational psychologist's work		
Neuropsychology	**0 courses**	**0 courses**	**0 courses**
Reviewed courses: N = 21			
Developmental psychology	**n: 1 course**	**0 courses**	**0 courses**
Reviewed courses: N =19	Direct – 0		
	Indirect/related – 1		
	Example:		
	Indirect/related: Parenting and attachment		
**Graduate Programs in Social Work**			
**University I**	**0 courses**	**n: 2 courses**	**n: 1 course**
Reviewed courses: N =38		Direct – 0	Direct – 0
		Indirect/related – 2	Indirect/related – 1
		Example:	Example:
		Indirect/related: Violence in the family	Indirect/related: Children at risk and out of home placement
**University II**	**n: 9 courses (8.6%)**	**n: 8 courses**	**0 courses**
	Direct – 1	Direct – 2	
Reviewed courses: N =104	Indirect/related – 8 (7.7%)	Indirect/related – 6 (5.8%)	
	Example:	Example:	
	Direct: Stress and suicide –	Direct: The theoretical basis for	
	treatment, prevention and education	interventions in traumatic situations	
	Indirect/related: Women's health: Psychological, behavioral and social aspects	Indirect/related: Children in trauma situations	
**University III**	**n: 5 courses (6%):**	**n: 5 courses (6%):**	**n: 2 courses**
		Direct – 0	Direct – 0
Reviewed courses: N =84	Direct – 2	Indirect/related – 5 (5.9%)	Indirect/related – 2
	Indirect/related – 3		
	Example:	Example:	Example:
	Direct: Parenting helps in preventing abuse of psychoactive substances	Indirect/related: Populations involved in risk behaviors	Indirect/related: Out of home placement of children and youth at risk
	Indirect/related: Stigma in the mental health field: Implications for the individual, the family and the community		
**University IV**	**n: 1 course**	**n: 2 courses**	**0 courses**
Reviewed courses: N = 42	Direct – 0	Direct – 0	
	Indirect/related – 1	Indirect/related – 2	
	Example:	Example:	
	Indirect/related: Drug policy	Indirect/related: Psychological processes in migration	
**Medical School**			
**University II**	**n: 1 course**		
Reviewed courses: N =95	Direct – 0		
	Indirect/related – 1	**0 courses**	**0 courses**
	Example:		
	Indirect/related: Epidemiology and preventive care		
**University III**	**n: 1 course**	**0 courses**	**0 courses**
Reviewed courses: N =108	Direct – 0		
	Indirect/ related – 1		
	Example:		
	Indirect/related: Growth and development		
**University VI**	**0 courses**	**n: 2 courses**	**0 courses**
Reviewed courses: N =168		Direct – 0	
		Indirect/related – 2	
		Example:	
		Indirect/related: Loss and grief	

^1^Direct primary prevention refers to interventions that can be applied directly (e.g., prevention programs for mental health problems); indirect/related primary prevention refers to studies providing information on modifiable factors that could be used ultimately in primary prevention interventions.^2^ Romanic numbers stand for name of a specified university.

Of the courses taught in graduate programs in social work, n = 35, 13.1%, addressed primary prevention. Most of them were electives, addressing universal and selective primary prevention concerning children and youth at risk, risk behavior and the impact of migration.

Finally, we reviewed courses offered in medical schools. Of the N = 367 courses, four indirectly addressed primary prevention in mental health.

### Master's and doctoral theses in psychology and social work programs

Of the N = 1960 approved doctoral and master's theses during the years 2007–2012, n = 123, 6.2%, addressed primary prevention in mental health (Table 3). The overwhelming majority of those addressed selective primary prevention indirectly, n = 106, 5.4% (e.g., "Loss and trauma: The impact of losing a teammate during battle on the psychological adjustment of soldiers who took part in the battle"). In addition, six theses, 0.3%, addressed issues related to universal primary prevention both directly and indirectly (e.g. “Primary prevention of eating disorders: Examination of the effectiveness of an experimental program for preventing an eating disorder at young ages"); and 11, 0.6%, addressed issues related to indicated primary prevention, often indirectly (e.g. “The predictive value of cognitive and behavioral characteristics for a psychotic episode among adolescents"). These doctoral and master's theses most commonly focused on the effects of traumatic events, particularly political events on mental health related issues among children and adolescents, the impact of migration on risk behaviors among youth, and risk factors for the development of eating disorders (Table 3).

**Table 3:** **Number and percentage of masters and doctoral theses in psychology and social work in universities in Israel addressing primary prevention, both direct and indirect** ^**1**^

Category	Indicated primary prevention	Selective primary prevention	Universal primary prevention
Masters and doctoral theses in psychology and social work departments in five universities in Israel.	n: 6 Masters and doctoral theses (0.36%):	n: 106 Masters and doctoral theses (5.4%):	n: 11 Masters and doctoral theses (0.6%):
Reviewed doctoral and master's theses: N = 1960	Direct – 2	Direct – 2	Direct – 1
	Indirect/related – 4	Indirect/related – 104 (5.3%)	Indirect/related – 10 (0.5%)
**Doctoral Theses in Psychology**			
**University I** ^**2**^	**0 Theses**	**n: 8 Doctoral theses (6.5%):**	**0 Theses**
Reviewed doctoral theses: N = 123		Direct – 1	
		Indirect/related – 7 (5.7%)	
		Examples:	
		Direct: The interaction between suicidal behavior and socio-cultural factors: A new examination of the	
		sequence approach compared with the categorical approach (part of the WHO Suicide Prevention project in Europe)	
		Indirect/related: Risk and resilience factors for the development of post-traumatic stress disorder among infants as a result of extended exposure to terrorism and warfare	
**University II**	**0 Theses**	**n: 1 Doctoral thesis**	**0 Theses**
Reviewed doctoral theses: N = 26		Direct – 0	
		Indirect/related – 1	
		Example:	
		Indirect/related: The relationship between difficult life experiences, subjective well-being, adjustment in aging and morbidity	
**University III**	**0 Theses**	**n: 1 Doctoral thesis**	**n: 1 Doctoral thesis**
Reviewed doctoral theses: N = 34		Direct – 0	
		Indirect/related – 1	
		Example:	
		Indirect/related: Genetic factors and personality traits associated with anorexia nervosa among female athletes and not-athletes	
			Direct – 0
			Indirect/related – 1
			Example:
			Indirect/related: The role of cognitions in the development of post-traumatic stress disorder
**University IV**	**0 Theses**	**n: 3 Doctoral theses**	**n: 2 Doctoral theses**
Reviewed doctoral theses: N = 35		Direct – 0	
		Indirect/related – 3	
		Example:	
		Indirect/related: Vulnerability to depression and somatization among Bedouin and Jewish students	
			Direct – 0
			Indirect/related – 2
			Example:
			Indirect/related: Early developmental pathways in Attention Deficit Hyperactivity Disorder (ADHD)
**University V**	**0 Theses**	**n: 6 Doctoral theses (16.6%):**	**0 Theses**
Reviewed doctoral theses: N = 36		Directed – 0	
		Indirect/related – 6 (16.6%)	
		Example:	
		Indirect/related: Psychological adjustment following the experience of combat: a comparative study among soldiers who were injured or lost their teammates during a combat	
**Master's Theses in Psychology**			
**University I**	**n: 2 Master's theses** Direct – 0	**n: 9 Master's theses** **(3.6%)**	**0 Theses**
Reviewed master's theses: N = 248	Indirect/related – 2	Direct – 0	
	Example:		
	Indirect/related: The relationship between major life events, personality and depression and anxiety among youth in the general population		
		Indirect/related – 9 (3.6%)	
		Example:	
		Indirect/related: The impact of social support, self-disclosure and life events on psychological distress, depression and suicidal tendency among Gush Katif evacuees groups during the Disengagement Plan	
**University II**	**n: 1 Master's thesis**	**n: 8 Master's theses** **(3%)**	**n: 2 Master's theses**
Reviewed master's theses: N = 267	Direct – 1 (0.4%)	Direct – 0	
	Indirect/related – 0	Indirect/related – 8 (3%)	
	Example:	Example:	
	Direct: Political violence and psychological distress: The impact of a school based intervention program on behavioral problems and post-traumatic stress disorder among adolescents	Indirect/related: Children of migrant workers in Israel: Self-esteem and self-efficacy as moderating factors in the relationship between minority stress and psychological distress and behavioral problems	
			Direct – 1
			Indirect/related – 1
			Example:
			Direct: Resilience in the face of political violence: A school based intervention program for youth at risk: Behavioral problems and social support
			Indirect/related: When the senses are too sharp: Sensory regulation, ritual and anxiety among preschoolers
**University III**	**n: 1 Master's thesis**	**n: 6 Master's theses** **(3.7%)**	**0 Theses**
Reviewed master's theses: N = 163	Direct – 1(0.6%)	Direct – 0	
	Indirect/related – 0	Indirect/related – 6 (3.7%)	
	Example:	Example:	
	Direct: Primary prevention of eating disorders: Examination of the effectiveness of an experimental program for preventing an eating disorder at young ages	Indirect/related: Predictors of emotional distress among delinquent adolescents residing in sponsored dormitories: individual and familial risk factors, and gender differences	
**University IV**	**0 Theses**	**n: 3 Master's theses**	**n: 5 Master's theses (3.3%):**
Reviewed master's theses: N = 153		Direct – 0	Direct – 0
		Indirect/related – 3	Indirect/related – 5 (3.3%)
		Example:	Example:
		Indirect/related – "You're abnormal if you're not afraid": Coping and resilience in the discourse of Israeli bus drivers who have experienced terrorist attacks	Indirect/related – Examining effortful control ability and negative emotionality in 2 & 3 years of age children at risk of ADHD
**University V**	**0 Theses**	**n: 7 Master's theses (2.5%)**	**0 Theses**
Reviewed master's theses: N = 278		Direct – 0	
		Indirect/related – 7 (2.5%)	
		Example:	
		Indirect/related: Loss and trauma: The impact of losing a teammate during battle on the psychological adjustment of soldiers who took part in the battle	
**Doctoral Theses in Social Work**			
**University I**	**0 Theses**	**n: 2 Doctoral theses**	**n: 1 Doctoral thesis**
Reviewed doctoral theses: N = 76		Direct – 0	Direct – 0
		Indirect/related – 2	Indirect/related – 1
		Example:	Example:
		Indirect/related: Loss of resources, personal values and development of distress among Israeli terror victims, natives and immigrants	Indirect/related: The predictive value cognitive and behavioral characteristics for a psychotic episode among adolescents
**University II**	**n: 1 Doctoral thesis**	**n: 2 Doctoral theses**	**0 Theses**
Reviewed doctoral theses: N = 22	Direct – 0	Direct – 0	
	Indirect/related – 1	Indirect/related – 2	
	Example:	Example:	
	Indirect/related: Gambling among teens: Association with temperament, coherence and exposure to advertisement	Indirect/related: Behavioral and emotional functioning of adolescents who were adopted at infancy and adolescents who were adopted after age two	
**University III**	**0 Theses**	**n: 6 Doctoral theses (14.3%)**	**0 Theses**
Reviewed doctoral theses: N = 42		Direct – 0	
		Indirect/related – 6 (14.3%)	
		Example:	
		Indirect/related: Exposure to violence in the community and its consequences among adolescents in Israel: Can family and teachers' support moderate the negative effects of the exposure?	
**University IV**	**0 Theses**	**n: 2 Doctoral theses (25%)**	**0 Theses**
Reviewed doctoral theses: N = 8		Direct – 0	
		Indirect/related – 2 (25%)	
		Example:	
		Indirect/related: Childbirth as re-traumatization: Childhood sexual abuse and its effects on symptoms of distress before and after giving birth	
**University V**	**0 Theses**	**n: 2 Doctoral theses**	**0 Theses**
Reviewed doctoral theses: N = 24		Direct – 0	
		Indirect/related – 2	
		Example:	
		Indirect/related – Pick and get hurt? The effect of working with battered women on their care providers' life	
**Master's Theses in Social Work**			
**University I**	**0 Theses**	**n: 5 Masters theses (7%):**	**0 Theses**
Reviewed master's theses: N = 71		Direct – 0	
		Indirect/related – 5 (7%)	
		Example:	
		Indirect/related: Grow and overcome: youth after the Second Lebanon War: The relationship among the characteristics of the exposure, the eviction from home and the school atmosphere on stress reactions a year after the war	
**University II**	**0 Theses**	**n: 10 Master's theses (7.6%):**	**0 Theses**
Reviewed master's theses: N = 131		Direct – 0	
		Indirect/related – 10 (7.6%)	
		Example:	
		Indirect/related: The relationship between the intergenerational conflict among Arab female adolescents and the tendency to develop an eating disorder	
**University IV**	**0 Theses**	**n: 13 Master's theses (18.1%):**	**0 Theses**
Reviewed master's theses: N = 72		Direct – 1	
		Indirect/related – 12 (16.7%)	
		Example:	
		Direct: Success factors in culturally sensitive interventions of *Maslan* – Rape Crisis Center in the Negev	
		Indirect/related: Consequences of political violence on mental, social and familial functioning of youth from the Gaza Strip and West Bank	
**University V**	**n: 1 Master's thesis**	**n: 12 Master's theses (8%):**	**0 Theses**
Reviewed master's theses: N = 151	Direct – 0	Direct – 0	
	Indirect/related – 1 (0.7%)	Indirect/related – 12 (8%)	
	Example:	Example:	
	Indirect/related: The relationship between eating disorders and body image and media exposure and sense of empowerment among adolescence in Israel	Indirect/related: Long-term effects of the Second Lebanon War on Adolescents: The connection between level of exposure and personal and social resources with post-traumatic symptoms, anxiety, and perception of the future	

^1^Direct primary prevention refers to interventions that can be applied directly (e.g., prevention programs for mental health problems); indirect/related primary prevention refers to studies providing information on modifiable factors that could be used ultimately in primary prevention interventions.

^2^Romanic numbers stand for name of a specified university 2007–2012.

## Discussion

We investigated the extent to which primary prevention in mental health is included in journal articles and academic programs in Israel. Our findings show that despite the application of a highly liberal definition of primary prevention, future mental health professionals are rarely offered specific courses on primary prevention, and when available, they are not mandatory. It is thus, not surprising that only a small fraction of the doctoral dissertations and master's theses in psychology and social work university departments addressed issues related to primary prevention. The scarcity of exposure to mental health primary prevention is reflected in the meager proportion of publications in the three leading journals we surveyed. In the recent decade, less than 10% of publications addressed issues related to primary prevention, mostly of an indirect application.

As noted earlier, we employed a liberal approach (at times, highly liberal) during the coding of the materials in this review. If we erred it was on the side of over-inclusiveness. Thus, it is likely that the numbers actually represent a somewhat over-estimation of primary prevention themes in education and research in mental health. Indeed, only 11 (4.7%) articles, 5 (0.6%) courses, and 5 (0.3%) doctoral and master's theses addressed primary prevention directly.

Our findings should be considered within recent developments that provide the ethical, clinical and human rights bases for a call for action to advance mental health primary prevention policy and activities in education, research and practice [7] as detailed below:

- Human rights issues. The right to health that has been enshrined in the WHO Constitution adopted in 1948 is stated as: “*The enjoyment of the highest attainable standard of health is one of the fundamental rights of every human being without distinction of race, religion, political belief, or economic or social condition”.* With the passing of time, two UN Conventions that were adopted by almost all the nations of the world, including Israel, lent greater strength to those principles. The more recent of those Conventions, on the Rights of Persons with Disabilities [11], is invoked generally with regard to curative care and rehabilitation. Yet, it also contains principles and articles that highlight the interconnectedness between human rights and primary prevention, such as prevention of abuse (article 16); respect for home and the family (article 23), education (article 24), and health (article, 25); as well as the adequate standard of living and social protection (article 28). Undoubtedly, it is the UN Convention on the Rights of the Child [12] that turns primary prevention into a chief issue, as illustrated by this excerpt: “*Recognizing that the child, for the full and harmonious development of his or her personality should grow up in a family environment, in an atmosphere of happiness, love and understanding*”. Therefore, the failure of the mental health system to meet the demands of the Conventions risks a violation of human rights.
- Recent epidemiological surveys conducted in Israel among adults [13] and youth [14], substantiate a solid case for mental health action. Those findings have shown that members of socially disadvantaged groups (e.g., ethnic minorities) are at higher risk for mental and behavioral disorders compared with members of advantaged groups [15, 16], thus creating a residue of vulnerable populations whose mental health risk may cross generations [17]. Importantly as well, studies have found that the treatment gap (i.e., the proportion of individuals with a diagnosable mental disorder who do not receive treatment) is high [18–20], including among vulnerable population groups [21, 22]. With regard to children, the treatment gap is even higher than among adults [23]. To compound this picture, stigma (the hidden burden of mental disorders [24]) and negative attitudes towards formal mental health care constitute concrete barriers that delay help-seeking or generate social exclusion [25, 26].
  A question thus arises, given the high burden of mental disorders and the wide treatment gap- could curative care suffice by itself to cover the needs or demands during the era of the Mental Health Reform? In our opinion, this set of factors overwhelms the possibilities of curative care making the inclusion of primary prevention a reasonable strategy of mental health action [7].
- Preventive programs for modifiable mental health problems do exist and receive growing attention. Take the case of child abuse, a true epidemics with immediate and long-term effects. The number of affected children by abuse is staggering. In 2010, U.S. state and local child protective services (CPS) received an estimated 3.3 million reports of children (43.8 per 1,000) being abused or neglected. The CPS estimated that 695,000 children (9.2 per 1,000) were substantiated victims of maltreatment. Of them, 78% were victims of neglect; and 18% of physical, 9% of sexual, and 8% of emotional abuses [27]. In Israel, in the year of 2009, 4,888 police records were initiated with regard to violence against minors [28]. Likely, the latter figures are an underestimate of events.
  Epidemiologic studies have shown that child or adolescent abuse have significant impact on children [29, 30] (e.g., externalizing behaviors, disruptive behavior, conduct and academic problems in school, depressive symptoms), and on adolescents (e.g., delinquent behavior, drug use, academic maladjustment, depression) [31]. In addition, there are late effects among adults, including: affective and anxiety disorders, suicide behavior, substance abuse disorders and even psychosis [32–35]. Importantly, mental health primary prevention action has been gaining solid scientific foundations as a result of the relatively recent Gene by Environment studies, with insights regarding risks [36, 37] and protective factors [38]. The existence of tested preventive programs open new opportunities for care, such as the Triple Parenting Program (PPT), purported to provide universal, indicative and selective primary prevention [39].
  In sum, there is a clear convergence of epidemiology and neurosciences with regard to the effect of abuse, a feature of the “toxic stress” highlighted by a recent report of the American Academia of Pediatrics. Based on cutting-edge research, the Academia has proposed a new route to bring about a change in the concern and practice of pediatricians [40, 41], which may be mimicked by mental health providers.
  Importantly as well, the costs of child abuse, based on US data, as well as the savings generated by preventive programs, as reviewed by leading health economists in the United Kingdom, provide a case illustration to the economic benefits of primary prevention programs. The US Center for Disease Control estimated that the average lifetime costs for abuse that did not end in death reached to over US$200,000 (in 2010 US dollars) per victim, and to close to US$1,300,000 per fatal maltreatment. The annual total lifetime cost for new cases of abuse was estimated to reach US$124 billion. Those costs referred to expenditures incurred by multiple agencies: special education, juvenile delinquency, mental health and health care, the adult criminal justice system, as well as lost productivity [42]. Net savings over the years have been identified in the UK as a result of the reduction of expenditures in the above-noted agencies for the care of abused children following the introduction of primary prevention programs. In the absence of the latter the outcome could have been conduct disorders. The saving is persuasive, for each English pound invested society saves eight [43].
  In reviewing the above, we are aware that the role of primary prevention in reducing the burden of mental health disorders could be questioned. Often, the mistaken argument is made that in the absence of etiological knowledge primary prevention lacks scientific legs to walk on. However, even if the case was so, experience has shown that prevention could precede etiology, witness Snow’s pump in London (before the vibrio cholera was identified as a cause). Also, no public health agent would suggest abstaining from action until a vaccine to prevent AIDS is developed. Until that is available, the use of condoms would continue to be promoted to prevent the transmission of the HIV virus.

### Limitations

Our inquiry has several limitations. First, although we established inter-rater reliability throughout the coding, some misclassifications may have occurred. Second, potential missing information may have occurred, particularly with regard to information obtained from academic institutions. Third, we reviewed curriculum in graduate programs in the fields of medicine, psychology and social work that train professionals responsible for the delivery of direct specialty care in mental health. Although other departments also have a role in the integrative care provided in mental health (e.g., nursing, occupational therapy) and thus their curriculum should be reviewed in future studies, it was beyond the preliminary scope of the current paper to include them. Similarly, other training experiences such as clerkship of medical students in psychiatry rotations, and clinical psychology field work are grounds for training of primary prevention. Future studies should include a systematic investigation of such experiences. Forth, in the current paper we reviewed three leading journals in mental health and social sciences in Israel. It is possible that there might be additional publications relevant to mental health primary prevention in other Israeli medical and social welfare journals as well as publications by Israeli authors on the subject in the international literature. Although reviewing all relevant journals is beyond the scope the current paper we sampled additional publications in other relevant journals: *Social Security* (*Bitahon Sochialy*, in Hebrew, 18 papers published in year 2012); *The Medicine* (*HaRefuah*, in Hebrew, 69 papers published in year 2012); *Israeli Medical Association Journal* (77 papers published in year 2012); *Conversations* (*Sichot*, in Hebrew, 77 papers published in years 2010–2013). Of the papers reviewed none addressed universal and indicated primary prevention, and only a marginal number addressed selective primary prevention indirectly (N = 3; 1.6%). This selective review confirms our findings with regard to the paucity of publications on primary prevention in mental health in Israeli journals.

## Conclusions and policy implications

The WHO comprehensive report on primary prevention [2] indicates that in order to facilitate the effective implementation of primary prevention interventions within and across countries, it is imperative to foster appropriate conditions that include national policies, partnerships between relevant stakeholders, capacity building and training to develop expertise, research supporting the development and implementation of effective programs and policies, and resources and infrastructures that facilitate policy-making, program development, provision of preventive services and others. In many countries such conditions are still not developed or are poorly implemented [2].

Furthermore, for many medical diseases, from infections to cancer and cardiovascular disorders, the greatest contribution to health has been through prevention rather than treatment. However, mental health prevention at a public health level has received less attention than individualized treatment efforts. Thus, there is a need for the development of effective universal preventive approaches to the common mental disorders at a population level [44, 45]. The majority of resources, available for mental health service provision in the West are allocated to the treatment or rehabilitation of those experiencing metal health disorders. In contrast, the promotion of mental health and the prevention of mental illness have been given relatively few resources and relatively little status [44, 46]. This is in spite of the observation that, prevention efforts, if successful, could substantially reduce the numbers needing relatively expensive therapeutic or rehabilitative work.

The mental health reform that is on its way in Israel calls for charting a new course of care [6]. To do more of the same will be self-defeating, the debt to society will remain outstanding, and human rights violations may continue. The intimate link that is envisioned for primary health care and mental health operating under a joint umbrella provides a unique opportunity to plan evidence-based services that will answer the mounting mental health needs of the population.

Such multidimensional reform should encompass several spheres of action [6]. Theses can include: (1) redirection of resources from mental hospital care to community mental health care; (2) humanizing care and promoting human rights; (3) aiming to treat not only to symptom reduction but also to promote quality of life; (4) including service users and their families in every mental health setting (hospitals, clinics, hostels etc.) and in a range of functions, including overall planning and priority setting, mental health education, and quality control; (5) redirecting training to expand the knowledge and skills necessary to practice mental health in the community that are not acquired in hospital based training; (6) Extending the care network to go beyond the specialist psychiatric services and including the service user and their families, community agents, and primary health care system.

To make cost-effective mental health primary prevention possible, a coordinated action is necessary given the interdisciplinary nature of primary prevention. Clearly, the task of coordination should become embedded within a national strategy of intra- and inter-sectorial [1], such as health, the environment, housing, social welfare, labor and employment, education, criminal justice and human rights protection.

In this scenario, new training opportunities must respond to the needs of policies, programs and services. National level initiatives to develop capacity and expertise for culturally sensitive evidence-based prevention of mental disorders and promotion of mental health should be developed. If needed, international support should be sought.

In research, coordination implies the design of multisite novel and replication studies to expand the pool of local knowledge as well as longitudinal studies to test the long-term impact of preventive interventions measured by the reduction of the incidence of mental health problems and of costs.

At least two major challenges can be identified in the implementation of such programs: (a) adequate consideration of cultural and context variables, which are essential components of any program to be applied in real-life situations in Israel, may complicate the task of dissemination of evidence-based interventions; (b) funding for primary preventive programs encounters barriers. First, these programs usually have a long-term outcome, this places them at a disadvantage in contrast to curative care that have near-term benefits. Second, the four health care systems in Israel will need to make room for those programs in their budgets.

Many years ago, in 1962, the late Leon Eisenberg noted that mental health primary prevention had yet to be fully included in the repertory of actions of the mental health services. For the title of his article, in which he strongly advocated for a change in policy whose time had arrived, he borrowed Hillel’s expression: “If not now –when?” [47]. Half a century later the expression remains valid.

## Authors’ information

Ora Nakash is a senior lecturer and director of the Center for Cross Cultural Clinical Research at the school of psychology at the Interdisciplinary Center (IDC), Herzliya. Her research interests include mental health disparities; Liat Razon is a project manager at the Center for Cross Cultural Clinical Research at the school of psychology, Interdisciplinary Center (IDC), Herzliya; Itzhak Levav is a professor at the department of community mental health at Haifa University. His research interests include health promotion and solidarity in healthcare system.
